# Purification and Characterization of a Highly Efficient Calcium-Independent α-Amylase from *Talaromyces pinophilus* 1-95

**DOI:** 10.1371/journal.pone.0121531

**Published:** 2015-03-26

**Authors:** Liang Xian, Fei Wang, Xiang Luo, Yu-Liang Feng, Jia-Xun Feng

**Affiliations:** State Key Laboratory for Conservation and Utilization of Subtropical Agro-bioresources, College of Life Science and Technology, Guangxi University, Nanning 530004, China; University of Huddersfield, UNITED KINGDOM

## Abstract

Alpha-amylase is a very important enzyme in the starch conversion process. Most of the α-amylases are calcium-dependent and exhibit poor performance in the simultaneous saccharification and fermentation process of industrial bioethanol production that uses starch as feedstock. In this study, an extracellular amylolytic enzyme was purified from the culture broth of newly isolated *Talaromyces pinophilus* strain 1-95. The purified amylolytic enzyme, with an apparent molecular weight of 58 kDa on SDS-PAGE, hydrolyzed maltopentaose, maltohexaose, and maltoheptaose into mainly maltose and maltotriose and minor amount of glucose, confirming the endo-acting mode of the enzyme, and hence, was named *Talaromyces pinophilus* α-amylase (TpAA). TpAA was most active at pH 4.0–5.0 (with the temperature held at 37°C) and 55°C (at pH 5.0), and stable within the pH range of 5.0–9.5 (at 4°C) and below 45°C (at pH 5.0). Interestingly, the Ca^2+^ did not improve its enzymatic activity, optimal temperature, or thermostability of the enzyme, indicating that the TpAA was Ca^2+^-independent. TpAA displayed higher enzyme activity toward malto-oligosaccharides and dextrin than other previously reported α-amylases. This highly active Ca^2+^-independent α-amylase may have potential applications in starch-to-ethanol conversion process.

## Introduction

Starch is a kind of macromolecule carbohydrate that is composed of glucose units connected by α-1,4-glycosidic bonds in linear chains and α-1,6-glycosidic bonds in branching points [1]. The enzymes that degrade starch are called amylases and are classified into four groups, i.e. exoamylases, endoamylases, debranching enzymes, and transferases [2]. The α-amylase (EC 3.2.1.1) is a type of endoamylase that mainly breaks the internal α-1,4-α-D-glucan bonds of the substrate [1,2], and is widely applied in biorefinery, detergent manufacturing, food, medicine, textile and paper industries [2–4].

Fossil oil is a kind of nonrenewable resource. One of the alternatives to fossil oil is ethanol, which is a clean and renewable fuel [5,6]. The production and use of ethanol as fuel have been well developed in several countries such as Brazil, the United States of America and Canada [7–9].

The traditional ethanol-producing industry that uses starch as a feedstock employs a separate hydrolysis and fermentation (SHF) process. This process includes three sequential steps: liquefaction, saccharification and fermentation. In the first step of liquefaction, the gelatinized starch is liquefied to malto-oligosaccharides by the Ca^2+^-dependent dextrinizing α-amylase from *Bacillus licheniformis* [10–12] or related species at about 90°C and near neutral pH, with the addition of Ca^2+^ to enhance the enzyme activity and thermostability of the α-amylase [10,13–16]. Recently, some Ca^2+^-independent α-amylases, which are active and stable at high temperature and near neutral pH, have been reported to be candidates for possible substitution of the commercial Ca^2+^-dependent dextrinizing α-amylases [17–21]. The application of these enzymes would eliminate the adverse effects of the addition of Ca^2+^, since Ca^2+^ accelerates the deterioration of industrial equipments by forming the precipitate calcium oxalate, which blocks pipes and heat exchangers, inhibits the isomerization of glucose, and accumulates in the end product in the fructose production industry [14,15]. In the second step of saccharification, the malto-oligosaccharides (containing approximately 12% maltotriose and approximately 55% malto-oligosaccharides with higher degree of glucose polymerization (DP) [13]) are hydrolyzed to glucose by glucoamylase from *Aspergillus niger* or related species at about 60°C and pH 4.0–5.0 [22,23]. In the third step of fermentation, the glucose is converted to ethanol by *Saccharomyces cerevisiae* at 30–35°C and pH 4.0–5.0 [13,22,24].

During the last decade, the saccharification step and fermentation step of the SHF process were combined by directly adding glucoamylase and yeast after the malto-oligosaccharides syrup had been cooled down to 30–35°C, so that the hydrolysis of malto-oligosaccharides to glucose by glucoamylase and the fermentation of glucose to ethanol were simultaneously conducted at 30–35°C and pH 4.0–5.0 [22]. This new process was called simultaneous saccharification and fermentation (SSF) process [22]. The SSF process was proven to be better than the SHF process [25,26] and was applied in the starch-to-ethanol industry [22,24,27].

Since the present used glucoamylases exhibit poor performance at 30–35°C due to their high temperature optima [28,29], a much larger amount of glucoamylase is added in the SSF step converting malto-oligosaccharides to ethanol than was required in the SHF process. To reduce the cost of using glucoamylase, some Ca^2+^-dependent α-amylases can be added to accelerate the hydrolysis of malto-oligosaccharides as they act synergistically with glucoamylase [10,30].

The α-amylases used currently in industry [10–12,30] and their Ca^2+^-independent possible candidates [17–21] exhibit poor performance in hydrolyzing the malto-oligosaccharides, with low specific and relative activity at 30–35°C and pH 4.0–5.0 due to their high temperature optima and neutral pH optima. Although several Ca^2+^-independent α-amylases have been reported to display high relative activity at 30–35°C [31–34], their low enzymatic activities toward malto-oligosaccharides and near neutral pH optima have limited their potential application in the SSF step of malto-oligosaccharides-to-ethanol conversion, which is conducted at 30–35°C and pH 4.0–5.0 [22,24,27].

In this study, we purified and characterized one α-amylase from the newly isolated *Talaromyces pinophilus* strain 1–95. This α-amylase showed collectively good properties as it is Ca^2+^-independent, has high specific and relative activity at 30–35°C and an optimal pH of 4.0–5.0, and exhibits higher enzyme activity than other reported α-amylases in hydrolyzing malto-oligosaccharides and dextrin.

## Materials and Methods

### Ethics statement

No animal or protected plants were involved in this study, and the ecosystem was not compromised by the collection of experimental samples. This study did not harm the natural environment or the health of humans.

### Materials

Microorganism-containing samples were collected from the suburbs of Wuzhou city (Guangxi Zhuang Autonomous Region, China). These samples included soil, rotten starchy materials, rotten fruits and plants, and fermented Chinese pickled vegetables.

Soluble cassava starch was bought from the Mingyang Biochemistry Co., Ltd. (Nanning, China), and other carbohydrates were all purchased from Sigma Co., Ltd (Saint Louis, USA). Unless otherwise stated, all chemicals used in this study were of analytical grade.

ÄKTA purifier 100 instruments, HiPrep 16/10 phenyl fast flow hydrophobic interaction chromatography (HIC) column, HiPrep Q XL 16/10 sepharose anion exchange chromatography (AEC) column, and Sephadex G25 HiPrep 26/10 desalting column were all purchased from GE Co., Ltd. (Uppsala, Sweden). High performance liquid chromatography instrument was purchased from Shimadzu Co., Ltd. (Kyoto, Japan). Hypersil NH_2_ column (4.6 mm×250 mm) was purchased from Dalian Elite Analytical Instrument Co., Ltd. (Dalian, China).

### Screening and identification of amylolytic microorganisms

The isolating medium used for isolation of amylolytic microorganisms contained (g/L): soluble starch 10, tryptone 5, K_2_HPO_4_ 3, (NH_4_)_2_SO_4_ 2.5, MgSO_4_·7H_2_O 0.2, CaCl_2_ 0.13, FeSO_4_ 0.0255, Trypan Blue 0.06, and agar 30, at pH 5.0. The liquid cultivation medium did not contain Trypan Blue or agar; other components were the same as in the isolating medium. To release the microorganisms in sample, one gram of sample was thoroughly ground and submerged in 50 mL of sterile distilled water, and then was shaken at 28°C and 180 rpm for 30 min. The suspension was serially diluted, and then 50 μL of each dilution were evenly spread onto one isolating medium agar plate. After cultivating at 28°C for 5 days, microorganisms with a clear halo around the colony were inoculated into the liquid cultivation medium (100 mL of medium in a 500-mL Erlenmeyer flask). After 5 days of cultivation at 28°C and 180 rpm, the amylolytic activity in the supernatant was measured at 30°C and pH 4.0 by using the dinitrosalicylic acid (DNS) method [35]. Finally, fungal strain 1–95 was found to display the highest amylolytic activity and was chosen for further study. Fungal strain 1–95 was preserved by dispersing the spores in 20% glycerol and storage at –80°C, and was deposited at the China General Microbiological Culture Collection Center under the accession number of CGMCC 2645.

The fungal strain 1–95 was identified by analyzing its morphological and molecular data. For morphological observation, strain 1–95 was three-point inoculated on Czapek-Dox agar plate in 9-cm petri dish and incubated in dark at 25°C and 37°C, and morphological and microscopic examinations (using Optical Microscope with 400 times magnification) were performed. The Czapek-Dox agar media contained (g/L): sucrose 30, NaNO_3_ 3, K_2_HPO_4_•3H_2_O 1.3, MgSO_4_•7H_2_O 0.5, KCl 0.5, FeSO_4_•7H_2_O 0.01, ZnSO_4_•7H_2_O 0.01, pH 7.0, agar 1.5% [36].

For molecular data analysis, the partial internal transcribed spacer (ITS) sequence, and the partial coding sequences of the β-tubulin gene and calmodulin gene of the fungal strain 1–95 were amplified using the corresponding primer sets of ITS1 and ITS4 [37], Bt2a and Bt2b [38], and CMD5 [39] and PoCMDR1 (5’-CGCCAATCGAGGTCATGACATGG-3’), respectively. The PCR procedure was according to that described by Lin et al. [40]. The amplified products were sequenced by Sangon Biotechnology Co., Ltd. (Shanghai, China), and then the sequences were analyzed using BLASTN online tool (http://blast.ncbi.nlm.nih.gov/Blast.cgi).

### Enzyme and protein assays

The amylolytic activity was determined by measuring the reducing sugars released by substrate hydrolysis. Before enzyme and protein assays, the culture supernatant of fungal strain 1–95 and the purified amylolytic enzyme solutions were desalted using a HiPrep 26/10 desalting column with deionized water as eluent. In the enzyme assay process, 0.35 mL of 0.1 M citrate-phosphate buffer with a preset pH value containing 1% (w/v) substrate was preheated at the preset temperature for 5 min, and then 0.05 mL of appropriately diluted enzyme solution was added and mixed well. After 10 min of incubation, the hydrolytic reaction was stopped by adding 0.8 mL of DNS reagent (containing (g/L): rochelle salt 200, NaOH 20, dinitrosalicylic acid 10, crystalline phenol 2, Na_2_SO_3_ 0.5). Then the mixture was incubated in boiling water for 5 min followed by cooling down to room temperature. The absorbance value of the mixture under 540 nanometer was read and the amylolytic activity was calculated using a glucose standard curve constructed by using DNS method [35]. One unit of enzyme activity was defined as the amount of enzyme required to release 1 μmol of glucose equivalents per minute under the assay conditions.

Protein concentration was determined via the BCA method [41] using a Micro BCA protein assay reagent kit (Pierce Co., Ltd., Rockford, USA). The molecular weight and purity of the protein were determined by sodium dodecyl sulfate—polyacrylamide gel electrophoresis (SDS-PAGE). Both SDS-PAGE and native PAGE were carried out using 5% stacking gel and 12% separating gel [42] with a Bio-Rad minigel system at 100 V and 4°C. A modified gel-staining water solution containing 0.25% Coomassie Blue R 250 (W/V), 45% methanol (V/V) and 10% acetic acid (V/V) [43] was used to stain the gel, and a simple gel-destaining water solution containing 20% acetic acid (V/V) was used to remove the background.

### Zymogram analysis

For zymogram analysis using native PAGE, denaturant and reductant were excluded in the electrophoresis system, and 1 g/L soluble starch was contained in the separating gel [44]. When the electrophoresis was completed, the gel was divided into two parts. One part was stained by gel-staining solution then de-stained by gel-destaining solution for observation of the protein band; the other part was incubated at 37°C for 1h and the amylolytic activity was observed by staining with a water solution containing 0.2% I_2_ (W/V) and 2% KI (W/V), a transparent zone appeared with a deep blue background indicating the location of amylolytic enzyme.

### Enzyme purification

The culture broth of fungal strain 1–95 displaying amylolytic activity was obtained by liquid cultivation using the method described above. The cell-free culture supernatant was gently mixed with a 33% volume of 4 M ammonium sulfate solution (pH 6.5) and incubated at 4°C for 24 h. After centrifugation at 8000×g for 10 min (4°C) and followed by filtration with a 0.22-μm filter membrane, the supernatant was applied to HIC using a HiPrep 16/10 phenyl fast flow column, and the bound proteins were eluted with a linear gradient of ammonium sulfate from 1 M to 0 M (in 0.02 M sodium phosphate buffer, pH 6.5). The eluted fractions displaying amylolytic activity were combined and desalted using a HiPrep 26/10 desalting column with 0.02 M Tris-HCl (pH 8.0) as eluent, and then subjected to AEC using a HiPrep Q XL 16/10 sepharose column. The bound proteins were eluted with a linear gradient of NaCl from 0 to 1 M (in 0.02 M Tris-HCl buffer, pH 8.0), and the eluted fractions displaying amylolytic activity were subjected to native PAGE and zymogram analysis to determine the protein band corresponding to the amylolytic enzyme. To obtain large amount of the amylolytic enzyme, the remaining eluent fractions containing the amylolytic enzyme were combined and subjected to native PAGE at 4°C for 4 h. After electrophoresis, the zone of unstained gel containing the protein band of the amylolytic enzyme, which was localized by native PAGE and zymogram analysis, was cut and ground, and then dispersed in 0.02 M Tris-HCl (pH 8.0) at 4°C for 24 h to allow the amylolytic enzyme to permeate into the buffer.

The enzyme solution was concentrated by using a 10 kDa cutoff ultra-filtration membrane, and then the purity of enzyme was determined by using SDS-PAGE. The purified enzyme was desalted and used for all the experiments described below.

### Analysis of the products of enzymatic hydrolysis of malto-oligosaccharides

The purified enzyme was used at a dose of 0.2 U/mg substrate (measured at pH 5.0 and 55°C, with soluble starch as substrate) to hydrolyze the linear malto-oligosaccharides with the degree of glucose polymerization from 3 to 7 (DP3 to DP7) at 30°C and pH 5.0 for 12 h. The hydrolysates were filtered through a 0.2-μm filter membrane and analyzed by high performance liquid chromatography (HPLC) using a Hypersil NH_2_ column. A water solution containing 74% acetonitrile (V/V) was used as the mobile phase with a flow rate of 1 mL/min. The hydrolysis products were detected using a RID-10A SHIMADZU refractive detector, with a solution containing glucose and DP2 to DP7 as standards.

### Effects of pH and temperature on the amylolytic activity and stability of the enzyme

The effect of pH on the enzyme activity was determined by measuring the enzyme activity at pH 3.0–7.0 (in 0.1 M citrate-phosphate buffer) and 37°C. The effect of temperature on the amylolytic activity was determined by measuring the enzyme activity at pH 5.0 and temperatures from 25°C to 75°C (with or without 5 mM CaCl_2_) [17]. The highest enzyme activity was defined as 100%, and other enzyme activities were calculated as relative activities.

The effect of pH on enzyme stability was determined by mixing 0.1 mL of enzyme solution with 0.9 mL of 0.1 M buffers with different pH values. The buffers used were citrate-phosphate buffer (pH 3.0–7.0), Tris-HCl buffer (pH 7.0–9.0), and glycine-NaOH buffer (pH 8.5–11.0). After incubating at 4°C for 24 h, the residual enzyme activities were measured at pH 5.0 and 55°C. The effect of temperature on enzyme stability was tested by incubating the enzyme at 40–65°C for 1 h (without substrate, with or without 5 mM CaCl_2_) [18,19,34], and the residual enzyme activities were measured at pH 5.0 and 55°C. The activity of the untreated enzyme was defined as 100%; other enzyme activities were calculated as relative activities.

### Kinetic parameters

The kinetic parameters of the α-amylase with respect to different soluble starches were measured at pH 5.0 and 55°C, with the concentration of the substrate ranging from 1.25 to 10 g/L, All experiments were performed in triplicate, and mean values were used to calculate the Km and Vmax values using the Lineweaver-Burk plotting method.

### Effects of metal ions and chelating reagents on the enzyme activity

The enzyme activity was measured at pH 5.0 and 55°C in the presence of different individual additives of CaCl_2_, CoCl_2_, CrCl_3_, CsCl_2_, CuCl_2_, FeCl_2_, FeCl_3_, KCl, LiCl, MgCl_2_, MnCl_2_, NaCl, NiCl_2_, Pb(NO_3_)_2_, ZnCl_2_, AgNO_3_, ethylenediaminetetraacetic acid (EDTA), disodium ethylenediaminetetraacetic acid (Na_2_EDTA), and ethylene glycol tetraacetic acid (EGTA). These additives were widely used in many previous reports of α-amylase, and thus were selected for this study. These additives were individually added into the enzyme assay system to reach final concentration of 1, 2.5, 5, and 10 mM, and then the enzyme assay was conducted. The enzyme activity without additives was defined as 100%, and the enzyme activities measured with different additives were calculated as relative activities.

### Effects of low-molecular-weight organic solvents on the activity of the enzyme

The organic solvents methanol, ethanol, ethylene glycol, acetonitrile, ethyl acetate, n-propanol, isopropyl alcohol, acetone, glycerol, and n-butanol were used to determine the effects of low-molecular-weight organic solvents on the enzyme activity at pH 5.0 and 55°C. The concentration of ethanol was 5, 10, or 15% (V/V), respectively. The concentration of other additives was 5% (V/V). The enzyme activity without additives was defined as 100%, and the enzyme activities measured with different additives were calculated as relative activities.

### Substrate specificity

Enzyme activities were measured toward different substrates at a concentration of 1% (w/v) at pH 5.0 and 55°C. The enzyme activity on soluble starch was defined as 100%, and the enzyme activities on other substrates were calculated as relative activities.

### Nucleotide sequence accession number

The partial ITS sequence, partial coding sequences of the β-tubulin gene and calmodulin gene of the fungal strain 1–95 were deposited into the GenBank database under accession numbers FJ795356, KP342453, and KP342454, respectively.

## Results and Discussion

### Screening of microorganisms for amylolytic activity and identification of the fungal strain 1–95

To obtain mesophilic and acidic amylolytic enzymes, amylolytic microorganisms were isolated from the 115 collected samples at pH 5 and 28°C. Among the 115 samples, 99 microbial strains showing a clear hydrolyzing halo around the colony were isolated and screened by inoculating into liquid cultivation medium at pH 5 and 28°C for 5 days. Among these microbial strains, a filamentous fungal strain 1–95 was found to produce the highest amylolytic activity of 1.49 U/mL (measured at 30°C and pH 4.0) in the culture broth and was selected for further study.

**Fig 1:**
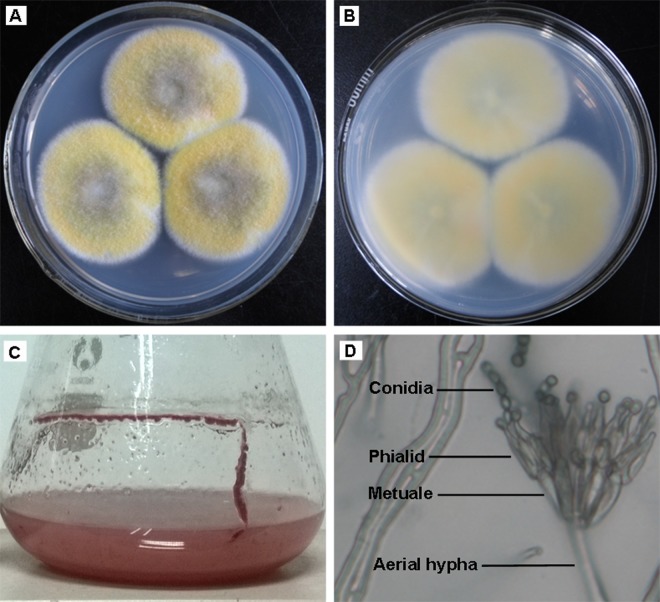
Morphological observation of fungal strain 1–95. Fungal strain 1–95 was cultivated using solid or liquid media, and then the morphological data were collected. (A) The obverse side of fungal strain 1–95. (B) The reverse side of fungal strain 1–95. (C) Fungal strain 1–95 produced red pigment when it was cultured using liquid isolating medium (containing 1% soluble starch). (D) The broom-like conidia generating structure and conidia of fungal strain 1–95.

The fungal strain 1–95 grew well at both 25°C and 37°C, but for ease of comparison with other strains, the morphological data for its growth at 25°C was described here. The newborn hyphae of fungal strain 1–95 were white, loose and floccose, and then turned yellow after 3 days of cultivation. Yellow color dominated the colony after 4 days cultivation, then the strain entered conidia generation phase, and the center of the colony began to turn green. After 9 days of cultivation, the diameter of the colony reached 4.5 cm (Fig. 1A). During these 9 days, the color of the reverse face of colony changed from white to golden brown (Fig. 1B). The colony had no conspicuous odor. As cultivation continued to 12 days, red pigment would be produced by the colony, and the red pigment was also produced after only 3 days of cultivation using the liquid isolation medium containing 1% soluble starch (Fig. 1C). Microscopic examination showed that the hyphae of fungal strain 1–95 were septate. The spore generation structure (including aerial hyphae, metuales and phialides) form a broom-like structure, with spore generation at the end of phialide (Fig. 1D), which is the distinctive characteristic of species belonging to *Talaromyces*. The conidia are globose to elliptical with smooth surface (Fig. 1D). The morphological data of fungal strain 1–95 are closely related to those of *Talaromyces pinophilus* (anamorph: *Penicillium pinophilus*) [45–47].

The ITS sequence of fungal strain 1–95 shared 100% identity with the ITS sequence of *Talaromyces pinophilus* (anamorph: *Penicillium pinophilus* [46]) (GenBank accession number AF176660) and 99.8% identity with the ITS sequences of five strains of *Talaromyces pinophilus* (GenBank accession numbers JQ776546, HM469418, GU566197, GU566200, and AB369480). The partial coding sequence of the β-tubulin gene of fungal strain 1–95 shared higher than 99% identity with those of the β-tubulin genes of eight strains of *Talaromyces pinophilus* (GenBank accession numbers JX091621, JX091381, KC992266, KM066145, KM066144, KM066143, KM066141, and KM066142). The partial coding sequence of the calmodulin gene of fungal strain 1–95 shared higher than 99% identity with those of the calmodulin genes of three strains of *Talaromyces pinophilus* (GenBank accession numbers AY678604, JX140697, and KF741964).

Based on the morphological and molecular data, fungal strain 1–95 was identified as *Talaromyces pinophilus*.

### Purification of an extracellular amylolytic enzyme from *T*. *pinophilus* 1–95

**Fig 2:**
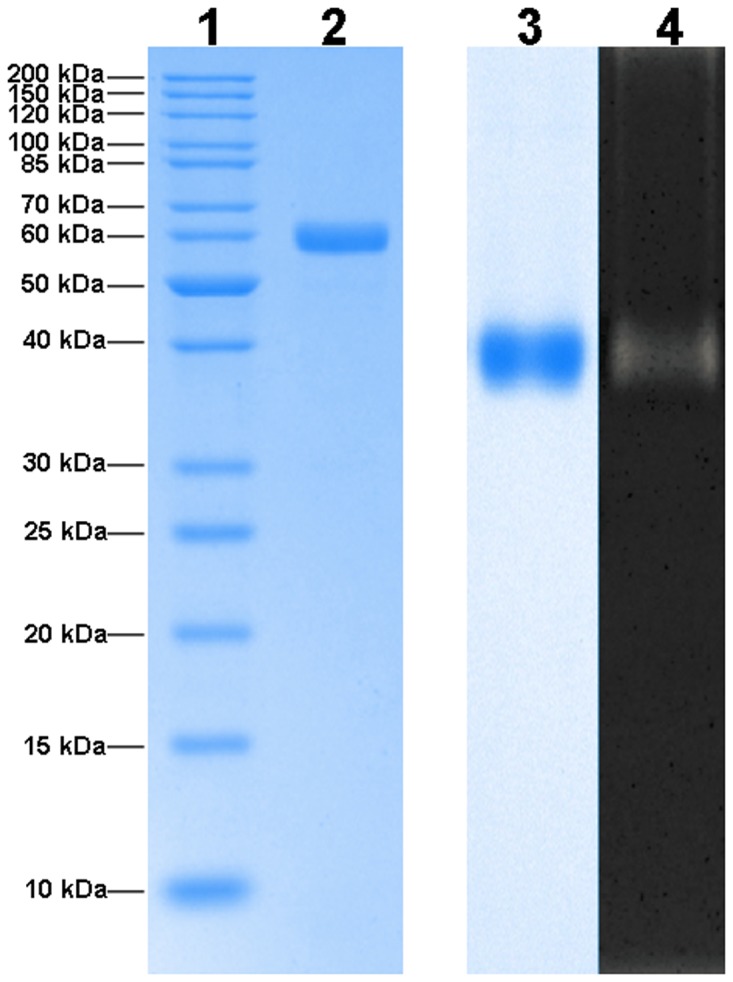
SDS-PAGE and native PAGE analysis of the purified amylolytic enzyme from *Talaromyces pinophilus* 1–95. Lane 1: protein molecular weight ladder on SDS-PAGE. Lane 2: the purified amylolytic enzyme from *Talaromyces pinophilus* 1–95 on SDS-PAGE. Lane 3: the purified amylolytic enzyme from *Talaromyces pinophilus* 1–95 on native PAGE. Lane 4: zymogram analysis of the purified amylolytic enzyme from *Talaromyces pinophilus* 1–95 on native PAGE.

As shown in Table 1, an extracellular amylolytic enzyme produced by *T*. *pinophilus* 1–95 was purified 130.77 folds from the liquid culture broth with a yield of 4.45%. The low yield was unavoidable because other proteins in some eluent fractions containing amylolytic enzyme had mobility similar to the amylolytic enzyme in native PAGE; these eluent fractions had to be discarded to avoid contamination of the purified enzyme. The purified amylolytic enzyme from *T*. *pinophilus* 1–95 displayed a single protein band on SDS-PAGE and native PAGE, and a single amylolytic band in separating gel of zymogram analysis (Fig. 2); these results confirmed the purity of the enzyme. Basing on the result of SDS-PAGE, the apparent molecular weight of the purified enzyme was estimated to be 58 kDa, which was similar to the α-amylases from *Bacillus licheniformis* NH1 (58 kDa) [48], *Bacillus* sp. KR-8104 (59 kDa) [20], *Bacillus* sp. TM1 (59 kDa) [31], and *Aspergillus niger* CBS 513.88 (63 kDa) [33]. The purified amylolytic enzyme from *T*. *pinophilus* 1–95 had a specific activity of 353.41 U/mg protein toward soluble starch at 30°C and pH 4.0.

**Table 1:** Summary of the purification procedure of an amylolytic enzyme from *Talaromyces pinophilus* 1–95.

Procedure	Total activity (U)	Total protein (mg)	Specific activity (U/mg)	Purification fold	Enzyme recovery (%)
Culture broth	3095.76	1145.48	2.70	1.00	100.00
(NH_4_)_2_SO_4_ precipitation	2480.65	518.59	4.78	1.77	80.13
HIC	1141.45	46.58	24.51	9.07	36.87
AEC	594.73	6.41	92.78	34.33	19.21
Native PAGE	137.83	0.39	353.41	130.77	4.45

### Hydrolysis of malto-oligosaccharides by the purified amylolytic enzyme from *T*. *pinophilus* 1–95

**Fig 3:**
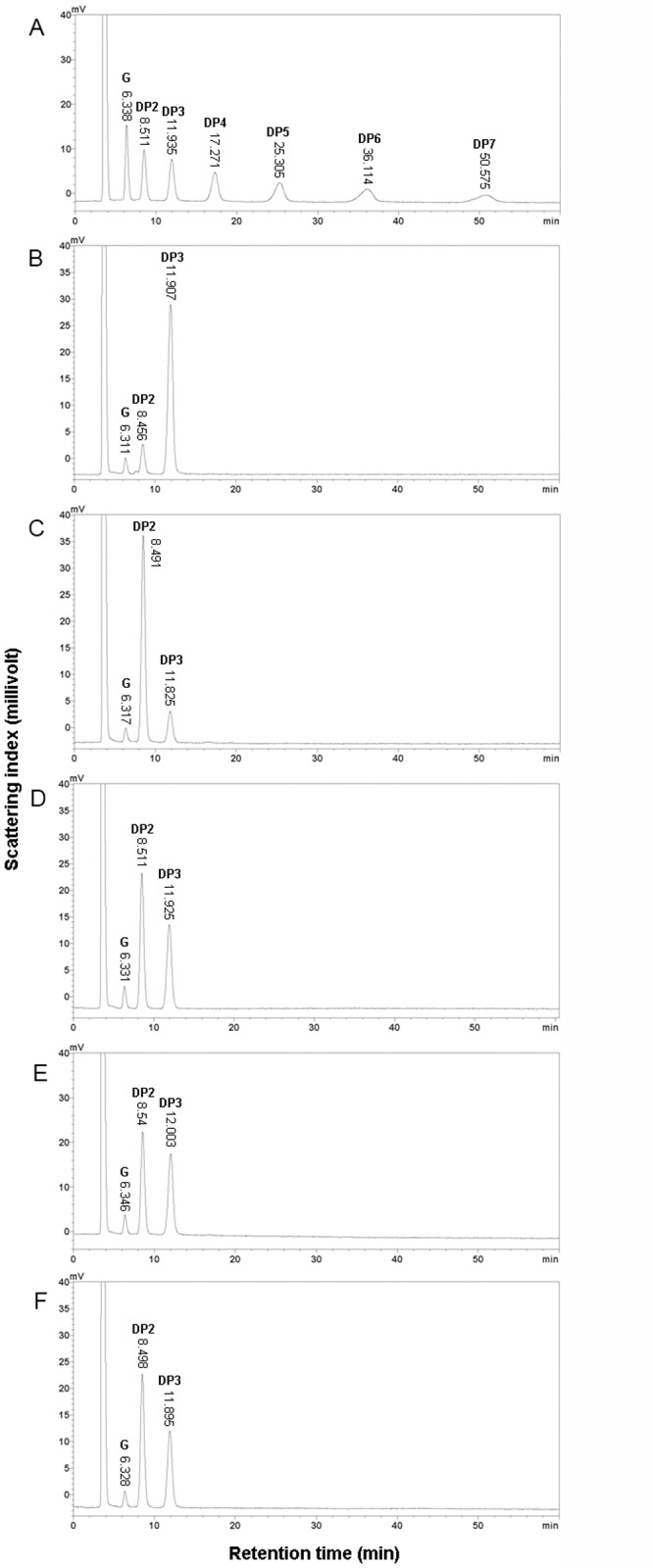
HPLC analysis of hydrolysates of malto-oligosaccharides produced by the purified amylolytic enzyme from *Talaromyces pinophilus* 1–95. The malto-oligosaccharides were hydrolyzed by the purified amylolytic enzyme at 30°C and pH 5.0 for 12 h at an enzyme dose of 0.2 U/mg substrate. The hydrolysates were analyzed by HPLC with a mixture of glucose, maltose (DP2), maltotriose (DP3), maltotetraose (DP4), maltopentaose (DP5), maltohexaose (DP6), and maltoheptaose (DP7) as standards. (A) Mixture of glucose, DP2, DP3, DP4, DP5, DP6, and DP7. (B) The hydrolysate of DP3. (C) The hydrolysate of DP4. (D) The hydrolysate of DP5. (E) The hydrolysate of DP6. (F) The hydrolysate of DP7.

By comparing their retention times to those of the standard sugars in HPLC (Fig. 3A), it was found that the purified amylolytic enzyme could partially hydrolyze maltotriose (Fig. 3B), and could completely hydrolyze DP4 releasing mainly maltose and trace amounts of glucose and maltotriose (Fig. 3C). The enzyme hydrolyzed DP5, DP6, and DP7 releasing mainly maltose and maltotriose, and a small amount of glucose (Fig. 3D, 3E, 3F). These results confirmed that the enzyme acted in the endo-hydrolyzing mode, thus was an α-amylase [1–3], and was named *Talaromyces pinophilus* α-amylase (TpAA).

In the ethanol-producing industry using starch as feedstock, a dextrinizing α-amylase is required in order to reduce the DP of starch at high temperature [13,23,24], and a saccharifying α-amylase is required in order to accelerate the complete hydrolysis of the malto-oligosaccharides to reduce the cost of using glucoamylase because it works synergistically with glucoamylase [10,30]. The α-amylase from *Penicillium expansum* [49] showed similar weak activity toward DP3 as TpAA. Unlike some α-amylases which could not hydrolyze DP3 [12,33], weakly hydrolyzed DP4 [48], or were strongly inhibited by the end products of DP2, DP3, and DP4 [18], TpAA showed superior ability to partially hydrolyze DP3 and completely hydrolyze DP4. Furthermore, the TpAA dose (0.2 U/mg substrate) used in this study was lower than those of some previously reported α-amylases [44,49–51]. Alpha amylase such as TpAA, which can efficiently hydrolyze malto-oligosaccharides to glucose, DP2 and DP3, may have potential industrial applications in that they accelerate the process by acting synergistically with glucoamylase [10,30].

### Effects of pH and temperature on the enzyme activity and stability of TpAA

**Fig 4:**
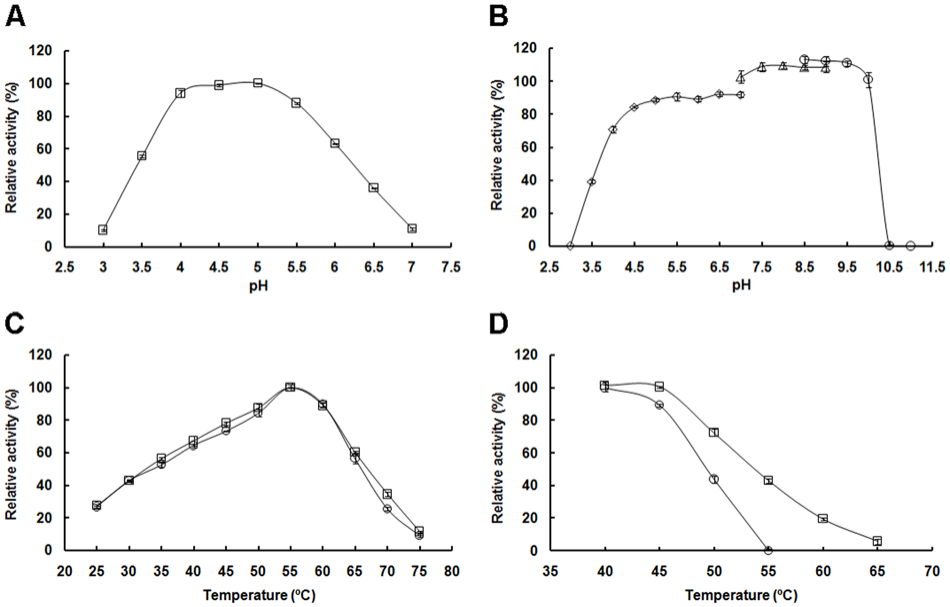
Effects of pH and temperature on the enzyme activity and stability of TpAA. (A) Effects of pH on the enzyme activity. The enzyme activities were measured at 37°C and pH 3.0–7.0 in 0.1 M citrate-phosphate buffer. (B) Effects of pH on the enzyme stability. The enzyme was mixed with 0.1 M buffers at pH 3.0–11.0 (pH 3.0–7.0, citrate-phosphate buffer; pH 7.0–9.0, Tris-HCl buffer; pH 8.5–11.0, glycine-NaOH buffer) and incubated at 4°C for 24 hours. The residue activities were measured at 55°C and pH 5.0. (C) Effects of temperature on the enzyme activity in the presence (○) or absence (□) of 5 mM CaCl_2_. The enzyme activities were measured at 25–75°C and pH 5.0 in the presence (○) or absence (□) of 5 mM CaCl_2_. (D) Effects of temperature on the enzyme stability in the presence (○) or absence (□) of 5 mM CaCl_2_. The enzyme was incubated at 40–65°C for 1 hour in the presence (○) or absence (□) of 5 mM CaCl_2_. The residue activities were measured at 55°C and pH 5.0. All values are expressed as percentages of the activity of untreated enzyme.

TpAA displayed pH optima within the range of 4.0–5.0 (Fig. 4A) and was stable within the pH range of 5.0–9.5 (Fig. 4B). It displayed temperature optima of 55°C (Fig. 4C) and was stable at temperatures below 45°C (Fig. 4D). It was also noted that the temperature optimum (Fig. 4C) and thermostability (Fig. 4D) of the enzyme were not enhanced by 5 mM CaCl_2_, indicating that the temperature optimum [17] and the thermostability [18,19,34] of TpAA were Ca^2+^-independent.

Alpha-amylases having different pH and temperature characteristics have different applications. Thermostable α-amylases are used to degrade gelatinized starch at high temperature [23,24,52], whereas detergent-stable and alkaline α-amylases are used in the washing industry [4,53]. Alpha-amylases acting well at low temperatures and acidic pH may have potential applications in the SSF step of the starch-to-ethanol conversion [10,26,30]. TpAA, which exhibited high relative activity and stability at pH 4.0–5.0 and 35°C, might be a potential enzyme candidate for applications in starch-to-ethanol processing at pH 4.0–5.0 and 30–35°C [22,24,27].

### Kinetic parameters

TpAA showed Km values from 2.51 to 6.70 mg/L toward six kinds of soluble starches (Table 2), which were similar to those of most previously reported α-amylases [17,34,44,54]. TpAA displayed Vmax values from 877.19 to 1051.52 U/mg for six kinds of soluble starches (Table 2). Moreover, the Vmax values of TpAA were higher than those of most reported α-amylases [17,34,55], and only lower than those of a few α-amylases, such as the Ca^2+^-dependent α-amylase from *Aspergillus oryzae* S2 [51]. The Km of TpAA toward dextrin from corn (linear glucose polymers with DP of approximately 10) was 2.34 mg/L, which was lower than that of McAA (6.40 mg/L) [21]; and the Vmax of TpAA toward dextrin was 684.93 U/mg, which was higher than that of McAA (175.4 U/mg) [21]. These results indicated that TpAA hydrolyzed dextrin more efficiently than McAA.

**Table 2:** Km and Vmax values of the TpAA to different soluble starches and dextrin.

	Soluble starch	Soluble starch (from corn)	Soluble starch (from wheat)	Soluble starch (from cassava)	Soluble starch (from rice)	Soluble starch (from potato)	Dextrin (from corn)
Km (mg/mL) ^a^	2.51	6.70	5.37	2.64	4.90	2.64	2.34
Vmax (U/mg) ^a^	918.27	1016.26	1051.52	900.90	949.67	877.19	684.93

^a^All experiments were performed in triplicate and mean values were used to calculate the Km and Vmax values using the Lineweaver-Burk plotting method. The experiments were repeated three times and similar results were obtained.

### Effects of metal ions and chelating reagents on the enzyme activity of TpAA

As shown in Table 3, 1, 2.5, 5, or 10 mM Ca^2+^ did not enhance the enzyme activity, indicating that the enzyme activity of TpAA was Ca^2+^-independent [17,20,32,56]. Further, 10 mM of Li^+^, Mg^2+^, and Na^+^ did not affect the enzyme activity, which was similar to BsAA [56]; 5 mM of Zn^2+^ did not affect the enzyme activity, which was similar to TLYAA [57]. The stimulating effect of 10 mM Cr^3+^ on the enzyme activity of TpAA was similar to that on BTMAA [31], and the stimulating effect of Co^2+^ on the enzyme activity of TpAA was similar to that on McAA [21] and AnAA [55]. The inhibitory effects of 10 mM Mn^2+^, Cu^2+^, Ag^+^, and Pb^2+^ on the enzyme activity of TpAA were similar to those on AtAA [54]. EDTA, Na_2_EDTA and EGTA had no effects on the enzyme activity of TpAA.

**Table 3:** Effects of metal ions on the enzyme activity of TpAA.

Inorganic metal salts	Relative activity^a^ (%)			
	Concentration (mM)			
	1	2.5	5	10
None	100.00±1.32	100.00±1.16	100.00±1.20	100.00±0.63
CaCl_2_	100.87±1.71	99.47±0.91	99.74±1.18	99.41±1.44
CoCl_2_	156.46±0.22	146.60±3.25	126.99±3.35	103.72±1.31
CrCl_3_	100.64±0.19	107.08±0.83	104.05±4.43	89.55±1.72
CsCl_2_	99.50±1.18	102.24±0.79	105.60±0.48	105.78±0.14
CuCl_2_	99.45±1.44	94.28±1.69	77.84±1.19	48.42±1.23
FeCl_2_	128.91±1.11	152.95±1.69	142.34±1.15	102.58±3.16
FeCl_3_	117.52±1.01	121.46±0.56	110.57±3.91	79.84±3.70
KCl	98.75±1.25	100.07±1.17	99.23±1.28	99.56±0.68
LiCl	99.83±0.36	98.07±0.78	97.68±0.23	97.28±0.58
MgCl_2_	97.99±0.29	98.25±1.59	99.74±0.80	99.15±0.51
MnCl_2_	96.91±0.89	88.39±2.66	75.63±1.54	60.01±1.16
NaCl	98.51±0.98	97.93±0.90	98.12±0.43	97.94±0.50
NiCl_2_	99.39±0.46	100.18±0.35	96.61±0.76	92.64±0.43
Pb(NO_3_)_2_	95.19±0.16	92.39±0.72	89.06±0.34	78.48±2.67
ZnCl_2_	99.21±0.11	102.00±3.44	101.55±3.74	101.21±5.37
AgNO_3_	98.22±0.27	88.11±2.36	77.03±1.64	61.30±4.44
EDTA	99.10±0.66	99.23±2.09	98.78±1.77	100.00±1.09
Na_2_EDTA	99.13±0.77	98.91±1.36	99.89±1.86	99.63±1.54
EGTA	100.41±0.76	97.02±2.49	100.48±0.41	99.67±0.63

^a^Data are means ± standard deviation from three replicates. The experiments were repeated three times, and similar results were obtained.

In previous reports, the effect of Ca^2+^ and chelating reagents on the activity of α-amylase varied. In general, Ca^2+^ stimulates the enzyme activity of or stabilizes α-amylases [10,16,30,51,55,57]. However, some α-amylases are Ca^2+^ insensitive [17,20,32,33,50,56]. Furthermore, in a few cases, Ca^2+^ inhibits the activity of α-amylase [21,31,58,59]. Although EDTA and Na_2_EDTA usually act as inhibitors of the activity of α-amylase [19,48,56,58], in a few cases, EDTA had been noted to stimulate the activity of α-amylase [31,59]. Nevertheless, some α-amylases had been reported to be insensitive to EDTA and Na_2_EDTA [20,33,44,50,54,55]. In the present study, TpAA was insensitive to Ca^2+^ and EDTA, similar to some α-amylases reported previously [20,33,50].

The enzyme activity of TpAA was insensitive to 10 mM Ca^2+^, Co^2+^, Cr^3+^, Fe^2+^, K^+^, Li^+^, Mg^2+^, Na^+^, or Zn^2+^; stimulated by 5 mM Co^2+^, Cs^2+^, Fe^2+^, or Fe^3+^; insensitive to 1 mM and only partially inhibited by 10 mM Cu^2+^, Mn^2+^, Pb^2+^, or Ag^+^. Overall, most of the tested metal ions at various concentrations had no effect on the enzyme activity or even stimulated the enzyme activity of TpAA, indicating that the enzyme activity of TpAA was highly compatible with these metal ions. Some α-amylases were reported to be poorly compatible with metal ions because their enzyme activities were seriously inhibited by most of metal ions mentioned above [21,31,51,56]. TpAA might be more suitable to be applied in the starch-to-ethanol process than these α-amylases [21,31,51,56], because metal ions will be present in the syrup derived from the starch-containing parts of plants (corn, cassava, potato, etc.) and used tap or river water.

### Effects of low-molecular-weight organic solvents on the enzyme activity of TpAA

TpAA exhibited relative activity of 91.84%, 84.08%, and 69.72% in the presence of 5%, 10%, and 15% ethanol, respectively, and showed high enzyme performance in the presence of 5% of other low-molecular-weight organic solvents (Table 4). This characteristic may contribute to the potential application of TpAA in the SSF step in the presence of gradually increasing concentrations of ethanol (up to 15% (V/V)) [27,60], n-butanol [61,62], acetone [61,62], or other low-molecular-weight organic solvents.

**Table 4:** Effects of low-molecular-weight organic solvents on the enzyme activity of TpAA.

Organic solvent	Relative activity^a^ (%)
None	100.00±0.81
Methanol	93.52±1.42
Ethanol (5%)	91.84±2.00
Ethanol (10%)	84.08±2.03
Ethanol (15%)	69.72±2.98
Ethylene glycol	91.21±2.94
n-Propanol	83.12±1.20
Isopropyl alcohol	93.46±1.10
Glycerol	114.66±2.88
n-Butanol	75.08±2.11
Acetonitrile	96.22±2.65
Acetone	97.60±1.40
Ethyl acetate	104.53±0.70

^a^Data are means ± standard deviation from three replicates. The experiments were repeated three times and similar results were obtained.

### Substrate specificity

TpAA showed higher activity toward amylose than amylopectin (Table 5), which might be due to the higher number of catalytic sites for an α-amylase in a linear substrate than in a branched one [63]. TpAA showed a high specific enzyme activity of 523.90 U/mg toward dextrin from corn. This activity was 2.3 folds of that of BsFAA (226.95 U/mg) [19] and fivefold that of McAA (103.44 U/mg) [21], but a comparison with other α-amylases cannot be made since these data were not included in most previous reports.

**Table 5:** Substrate specificity of TpAA.

Substrate	Specific activity^a^ (U/mg)	Relative activity^a^ (%)
Soluble starch	673.08±6.59	100.00±0.98
Soluble starch (from corn)	627.60±11.58	93.24±1.72
Soluble starch (from wheat)	635.70±10.88	94.45±1.62
Soluble starch (from cassava)	688.30±8.14	102.26±1.21
Soluble starch (from rice)	632.80±7.27	94.01±1.08
Soluble starch (from potato)	656.88±14.74	97.59±2.19
Amylose	712.76±9.35	105.90±1.39
Amylopectin	459.65±8.40	68.29±1.25
Dextrin	523.90±14.31	77.84±2.13
γ-Cyclodextrin	378.00±5.03	56.16±0.75
β-Cyclodextrin	ND	ND
α-Cyclodextrin	ND	ND
Pullulan	44.16±3.22	6.56±0.48

^a^Data are means ± standard deviation from three replicates. The experiments were repeated three times and similar results were obtained.

ND: Enzyme activity was undetectable toward this substrate.

TpAA could not hydrolyze α-CD (Cyclodextrin) and β-CD but could hydrolyzed γ-CD. This might be due to the cyclical shape of CD contributing to the configuration of substrate and making the substrate low accessibility to the active site of the enzyme, thus making enzyme catalysis difficult. The cyclical shape of CD with longer chain showed weaker effect on the configuration of the molecule than other CD with shorter chain. McAA displayed similar behavior with a high activity toward γ-CD and lower activity toward β-CD than γ-CD, and almost no activity toward α-CD [21]. Many α-amylases show a substrate bias of amylose > soluble starch > amylopectin and display no activity toward α-CD and β-CD [19,54].

TpAA exhibited very low activity toward pulullan, perhaps because TpAA prefers α-1,4 glycosidic bond to α-1,6 glycosidic bond, and the abundance of α-1,6-D-glycosidic bonds contributes to the configuration of the substrate and makes enzyme catalysis difficult [44,63]. Most other α-amylases showed weak activity toward pullulan [44,54], except for McAA [21], which showed high activity toward pullulan.

TpAA showed high specific activity toward soluble starches from corn, wheat, cassava, rice, and potato, and the highest enzyme activity was observed when cassava starch was the substrate. It is well known that corn, wheat, and cassava starches are widely used for fuel ethanol production [5–9]. The demand for cassava starch keeps increasing since industrial applications of cassava starch has multiple advantages [64]. Thus, a high activity toward corn, wheat, and cassava starches highlights the potential industrial applications of TpAA. A comparison of multiple enzymatic characteristics between TpAA and previously reported α-amylases was given in Table 6. Compared with other Ca^2+^-independent α-amylases, TpAA displays excellent comprehensive characteristics.

**Table 6:** Comparison of enzymatic characteristics among TpAA and previously reported α-amylases.

Source strain of AA	Ca^2+^-dependence (enzyme activity)	Ca^2+^-dependence (thermostability)	Temperature optimum (°C)	pH optimum	Specific activity toward soluble starch (U/mg)	Specific activity toward dextrin (U/mg)	Reference
***Talaromyces pinophilus* 1**–**95**	**independent**	**independent**	**55**	**4.0**–**5.0**	**673.08** ^**a**^	**523.90** ^**a**^	**This study**
*Aspergillus niger* CBS 513.88	independent	independent	37	6.0	2.5^**a**^	NM	[33]
*Bacillus* sp. DR90	independent	independent	45	4.0	780.79^**a**^	NM	[34]
*Bacillus* sp. Ferdowsicous	independent	independent	70	4.5	267^**a**^	226.95	[19]
*Bacillus* sp. KR-8104	independent	independent	75–80	4.0–6.0	330^**a**^	NM	[20]
*Thermococcus* sp. HJ21	NM	independent	95	5.0	8.3^**a**^	NM	[18]
*Aspergillus tamarii*	independent	NM	55–65	4.5–7.0	778.3^**a**^	NM	[54]
*Malbranchea cinnamomea* S168	independent	NM	65	6.5	514.6^**a**^	103.44^**a**^	[21]
*Meretrix lusoria*	independent	NM	40	6.5	2798.5^b^	NM	[32]
*Meretrix lusoria*	independent	NM	50	7.5	4711^b^	NM	[32]
*Bacillus* sp. GRE1	independent	NM	70	6.0	216.3^**a**^	NM	[17]
*Bacillus licheniformis* NH1	independent	dependent	90	5.0–10.0	178.5^**a**^	NM	[48]
*Bacillus subtilis* A28	independent	dependent	70	6.0	2814^**a**^	NM	[56]
*Aspergillus oryzae* S2	dependent	dependent	50	5.6	5089.3^**a**^	NM	[51]
*Aspergillus oryzae* S2	dependent	dependent	50	5.6	6686.3^**a**^	NM	[51]
*Lipomyces kononenkoae* IGC4052B	dependent	NM	40	4.0	1633^**a**^	NM	[44]
*Bacillus licheniformis* ATCC 9945a	dependent	dependent	90	6.5	3666.7^**a**^	NM	[11]
*Bacillus licheniformis* 44MB82-A	dependent	dependent	90	6.0–6.5	4300^**a**^	NM	[12]
*Thalassobacillus* sp. LY18	dependent	NM	70	9.0	151.8^**a**^	NM	[57]

^a^The enzyme activity was measured using DNS method.

^b^The enzyme activity was measured using iodine method, thus the result can’t be directly compared with those measured using DNS method.

NM: Not mentioned in the reference.

## Conclusions

In this study, TpAA was purified from a newly isolated filamentous fungal strain, *Talaromyces pinophilus* 1–95, and biochemically characterized. The enzyme activity and thermostability of TpAA were both found to be Ca^2+^-independent. When compared with other Ca^2+^-independent α-amylases, TpAA displays excellent comprehensive characteristics including high activity at pH 4.0–5.0 and high specific and relative activities at 30–35°C; high enzyme activity toward malto-oligosaccharides, dextrin, and soluble starches from corn, wheat, cassava, rice, and potato; and high compatibility with metal ions and insensitive to low-molecular-weight organic solvents. Thus, TpAA has potential applications in the SSF of starch to ethanol.
